# Morphometric study of the acetabulum among north Indian population: A cross-sectional radiological study

**DOI:** 10.6026/973206300214162

**Published:** 2025-11-15

**Authors:** Vibhash Kumar Vaidya, Vinod Kumar, Amit Kumar Yadav, Sudhakar Viswas, Akhil Sathyan, Swati Bang

**Affiliations:** 1 Department of Anatomy, Varun Arjun Medical College & Rohilkhand Hospital, Banthara, Shahjahanpur, Uttar Pradesh, India; 2 Department of Orthopaedic, Maharaja Suheldev Autonomous State Medical College, Bahraich, Uttar Pradesh, India; 3 Department of Anatomy, Maharaja Suheldev Autonomous State Medical College, Bahraich, Uttar Pradesh, India; 4 Department of Physiotherapy, National Institute of Management Technology, Greater Noida, Uttar Pradesh, India; 5 Department of Anatomy, ESIC Medical College & Hospital, Faridabad, Haryana, India

**Keywords:** Acetabulum, angle of sharp, acetabular dysplasia, Central Edge Angle (CEA), hip bone, morphometric variations, osteoarthritis (OA)

## Abstract

There is a need to estimate acetabular morphometric parameters in asymptomatic individuals from the North Indian population. Therefore, it is of interest to evaluate the prevalence of acetabular dysplasia, which has not been extensively studied in this specific population. A cross-sectional analysis of 100 radiographs was conducted to measure parameters such as center-edge angle, acetabular angle and acetabular depth. The data revealed gender-based differences in acetabular parameters, with significant variations noted between the right and left sides. Thus, we show valuable data for improving hip implant design, understanding osteoarthritis and supporting clinical and forensic applications. This study contributes to more precise and population-specific orthopedic interventions.

## Background:

The erect posture of humans is unique among primates and is maintained by large muscles in the vertebral column, gluteal and abdominal regions, along with structural adaptations in the vertebral column, hip, pelvis and blood vessels [1]. The center of gravity lies near the hip joint, with the line of gravity slightly anterior to the ankle joint [2]. Bipedalism, exclusive to humans, has led to biomechanical inefficiencies and conditions such as low back pain, sciatica, acetabular dysplasia, coxarthrosis, gonarthrosis and varicose veins [3]. Understanding these conditions requires knowledge of postural parameters of the acetabulum, including its shape, depth and variations due to age, sex and congenital morphology [4]. The acetabulum, a hemispherical cavity on the lateral aspect of hip bone, faces antero-inferiorly and has an irregular margin with an inferior deficiency at the acetabular notch. Its central floor forms the rough, non-articular acetabular fossa, while the anterosuperior lunate surface (dome) transfers weight to the femur [5]. It is composed of contributions from the pubis (anterosuperior fifth), ischium (fossa floor and postero-inferior two-fifths) and ilium (remaining two-fifths). Occasionally, a linear defect may traverse its surface [6]. Dysplasia is an abnormal cells stages or pre-cancerous cells that can lead to enlarged tissue. Skeletal dysplasia is responsible for the disorders known as dwarfism, brittle-bone disease and cherubism. There are about 350 disorders of the skeleton which are classified as dysplasia [7]. They are caused by a genetic mutation and generally diagnosed in infancy. In children, it is called developmental dysplasia of the hip (DDH) [8]. Acetabular dysplasia occurs when the acetabulum is shallow, vertically oriented and underdeveloped, reducing the weight-bearing contact area with the femoral head. This increases force per unit area during movement, raising the risk of early osteoarthritis [9]. The center-edge angle (CEA), first described by Wiberg in 1939, is a key diagnostic measure, CEA is the angle between a vertical line through the femoral head center and another line extending from the same center to the acetabulum's upper lateral margin. A CEA >25 degrees is normal, 20-25 degrees is borderline and <20 degrees indicates acetabular dysplasia [10]. The mean CEA for males is 37 degrees and for females, 35 degrees. However, CEA measurements can be inaccurate due to pathological femoral head conditions, osteophytosis, or hip subluxation [11]. Hence, acetabular angle and depth are also used to diagnose dysplasia. The acetabular angle, described by Sharp, is formed between lines connecting the pelvic teardrop, with normal values between 33-38° [12]. Acetabular depth refers to the measurement from the acetabular roof to a line connecting the lateral acetabular edge and the upper portion of the pubic symphysis. A depth of less than 9 mm is indicative of acetabular dysplasia [13]. The anatomical characteristics of the hip joint hold significant value for orthopedic specialists, radiologists and prosthetists. These parameters play a crucial role in designing patient-specific implants and contribute to a deeper understanding of the etiopathogenesis of osteoarthritis [14]. Additionally, insights into the normal dimensions of the acetabulum and gender variations can aid forensic experts in early and accurate sex determination in disputed cases [15]. Therefore, it is of interest to analyze normal acetabular morphometry, identify sex-based differences in anatomical parameters among asymptomatic individuals without structural abnormalities and assess the prevalence of acetabular dysplasia within the North Indian region.

## Methodology:

The present cross-sectional study analyzed 100 plain radiographs capturing the anteroposterior (AP) view of both hip joints in adult subjects. The present study was conducted in the Anatomy Department of Varun Arjun Medical College & Rohilkhand Hospital, Banthra, Shahjahanpur and Maharaja Suheldev Autonomous State Medical College, Bahraich. The X-ray images were obtained from the Orthopedics and Radiology Departments of Maharaja Suheldev Autonomous State Medical College, Bahraich. The study was carried out over six months, from August 2024 to January 2025. Plain radiographs from individuals aged 30 to 60 years, accessible in the Orthopedics and Radiology Departments at Maharaja Suheldev Autonomous State Medical College, Bahraich. X-ray images of the hip joint exhibiting pathological conditions were analyzed, while cases with a joint space width of less than 2mm, pregnancy, incomplete acetabular fusion, or a history of post-traumatic injury were excluded from the study. Using a convenient sampling approach and referencing, the minimum required sample size was determined. The calculation follows the formula. The sampling formula is N=z2αxpxq/L2 where N is sample size; p is percentage; q=1-p, Type of error α=5%, Allowable error L= 15% of p. So, estimated sample size calculated was 100. The specimens were anonymised, randomly coded and de linked from any identity sources (ICMR National guidelines for biomedical and health research involving human participants, ICMR, 2017, sec 5, Box 5.2) [16]. After obtaining written and informed consent, an anteroposterior (AP) radiograph was captured with the patient lying supine, with the leg fully extended and internally rotated at 15 degrees. The X-ray source was positioned 100 cm from the radiographic film, ensuring perpendicular beam alignment with the cassette. The X-rays were directed to enter 5 cm above the pubic symphysis.

Linear measurements were conducted by referencing the graduated scale visible on the radiograph shown in (Figure 1 - see PDF). All morphometric parameters were measured twice to minimize error and the average value was recorded (Figure 2 - see PDF). The collected data was tabulated and analyzed using SPSS software version 21.0. The maximum, minimum and mean values, along with standard deviation, were calculated for each percentage of the studied parameters. These parameters were compared between the right and left hip joints in males and females, with statistical analysis performed using an unpaired Student's t-test. A p-value of ≤ 0.05 was considered statistically significant.

## Results:

The present observational study analyzes three acetabular morphometric parameters to assess acetabular dysplasia prevalence in the North Indian population. It also examines sex differences and compares the morphometry of the right and left hips. The mean values of morphometric parameters, irrespective to their sides, showed that the Central Edge Angle (CEA) in males was 37.8 ± 5.5° on the right and 39.9 ± 6.8° on the left, while in females; it was 36.3 ± 7.6° on the right and 38.4 ± 6.7° on the left. The acetabular angle (AA) in males measured 36.2 ± 3.4° on the right and 36.5 ± 3.4° on the left, whereas in females, it was 37.6 ± 4.3° and 38.3 ± 2.8°, respectively. Acetabular depth (AD) in males was 14.4 ± 3.5 mm on the right and 14.8 ± 3.7 mm on the left, while in females, it was 14.1 ± 3.6 mm and 14.7 ± 3.2 mm, respectively. The maximum, minimum, mean, standard deviation, t-value and p-value (p ≤ 0.05 considered significant) for each parameter are presented in Table 2 (see PDF). A significant difference was observed in male CEA and AD on both sides (p = 0.002 and 0.05, respectively), as well as in female CEA and AD (p = 0.01) (Table 1 - see PDF). CEA ≤20° occurred in 2% overall, exclusively in females (4%) on the right. AD ≤9mm was seen in 9% of right side (6% males, 12% females) and 6% of left side (4% males, 8% females). AA ≥47° appeared in 2% of right side (4% females) and 1% of left side (2% females). No males had CEA ≤20° or AA ≥47° (Figure 3 - see PDF).

## Discussion:

The center edge angle, first described by Wiberg in 1939, is a key radiographic measurement used to differentiate normal hips from dysplastic ones. It assesses the coverage of the femoral head by the acetabulum, with values >25° indicating normal, 20-25° borderline and <20° suggesting dysplasia [17]. Acetabular depth, described by Murray, measures the distance from the acetabular roof to a line joining the lateral acetabular margin and upper pubic symphysis. A depth <9 mm points to dysplasia. The acetabular angle, described by Sharp, is formed between lines connecting the pelvic teardrop, with normal values between 33-38° [18]. The anatomical characteristics of the hip joint hold significant value for medical professionals across various fields, including orthopedics, radiology and prosthetics. These parameters play a crucial role in crafting patient-specific implants and provide insight into the underlying causes of osteoarthritis. Understanding the standard measurements of the acetabulum and their variations between genders is also essential for forensic experts in determining disputed sex at an early stage [19]. Over time, numerous anatomists and anthropologists have meticulously studied the dimensions of the femoral head and acetabulum. Their research highlights that the size of the femoral head is not fixed; rather, it fluctuates based on factors such as sex, ethnicity, genetic predisposition, climate and broader geographical influences [20]. The central edge angle of Wiberg, which indicates acetabular coverage of the femoral head, varies across populations. The lowest values are observed in South Korean females (25.6±6.2) [21] and Nigerian males (26.57±0.74) [22], while the highest values are reported in Serbian males (48.42±7.91) [23]. Acetabular angles of Sharp, which reflect the orientation of the acetabulum, range from approximately 35° to 44°, with the highest values recorded in Japanese and South Korean populations [24]. Acetabular depth, representing the depth of the hip socket, is inconsistently reported across studies, with values as high as 26.47 mm in Serbian males [25] and as low as nearly 0.28 mm in South Korean females [26]. The present study focuses on a North Indian population (n=100) and reports gender- and side-specific values for all three parameters. The central edge angle of Wiberg in North Indian male's ranges from 37.8° to 38.4°, while in females, it ranges from 36.3° to 38.4°. The acetabular angle of Sharp varies between 36.2° and 38.3° across genders and sides. The acetabular depth is reported to be around 14.1-14.8 mm, which is comparable to our findings. According to Padmakaran *et al.* (2023) [27], the study examines the various factors influencing acetabular morphology in different populations, highlighting significant regional differences in acetabular measurements. Their findings suggest that geographic and ethnic factors may contribute to variations in hip joint development, which is essential for orthopedic evaluations and surgical interventions. Khurana *et al.* (2023) [28] investigated the role of acetabular measurements in neonatal hip development, with a focus on gender-specific variations. Their study offers valuable insights into the early detection of hip dysplasia, emphasizing the need for standardized measurements in neonatal orthopedic care.

## Conclusion:

The study provides valuable morphometric data on the acetabulum specific to North Indian populations, offering insights for improved, population-specific hip implants. This data can guide orthopedic surgeons, prosthetists and radiologists in enhancing the accuracy of hip replacements and diagnosing osteoarthritis. Additionally, it holds forensic value in identifying sex, race and stature in disputed cases.
